# Reducing Exposure to High Fluoride Drinking Water in Estonia—A Countrywide Study

**DOI:** 10.3390/ijerph110303132

**Published:** 2014-03-14

**Authors:** Ene Indermitte, Astrid Saava, Enn Karro

**Affiliations:** 1 Department of Public Health, University of Tartu, Ravila 19, Tartu 50411, Estonia; E-Mail: astrid.saava@ut.ee; 2 Department of Geology, University of Tartu, Ravila 14a, Tartu 50411, Estonia; E-Mail: enn.karro@ut.ee

**Keywords:** high-fluoride exposure, drinking water, reduction measures, Estonia

## Abstract

Fluoride is a naturally occurring contaminant in groundwater in Estonia. There are several regions in Estonia with fluoride contents in public water supplies as high as 7 mg/L. Long-term exposure to high-fluoride drinking water may have several adverse health effects, primarily dental fluorosis. The opportunities for exposure reduction rely highly on water treatment technologies. Since 2004 public water suppliers in Estonia have made efforts to diminish fluoride content in drinking water systems. A follow-up study on a country level was carried out in 2004–2012 to analyze the changes in population exposure to excessive (over 1.5 mg/L) fluoride in drinking water and to get information about the reduction methods applied by public water supplies (PWS) to optimize the fluoride levels in public water system. The results showed that bigger PWS have been more effective in fluoride reduction measures than small PWS. The main methods used to lower the fluoride content were reverse osmosis technology and replacement of water sources with new ones (new drilled wells). As a result of all the measures taken the overall high-fluoride exposure has been reduced substantially (82%).

## 1. Introduction

Elevated levels of fluoride in drinking water (*i.e.*, levels above the World Health Organization guideline value of 1.5 mg/L) have been identified in numerous countries around the World [1,2], including Estonia [3]. Waters high in fluoride tend to be found in discrete areas, mostly in sodium-, potassium- and chloride-rich and calcium-poor groundwaters in many basement aquifers [4,5]. In Estonia elevated levels of fluoride (up to 7 mg/L) are found in the Silurian-Ordovician aquifer system, which is an important and often the only source of drinking water in central and western Estonia. The dissolution of fluorides from carbonate rocks and clayey K-bentonite beds is the natural source of fluoride [6,7].

Fluorides in small quantities have a practical role in the protection against dental caries [8]. However, its excessive intake may result in several adverse health effects. The first sign of fluoride toxicity is dental fluorosis [9,10]. Besides dental fluorosis, chronic excessive consumption of fluoride may lead to skeletal fluorosis [10] and hip fractures among the elderly [11]. Fewtrell *et al*. have estimated that high fluoride concentrations in drinking water have caused about 47 million of dental fluorosis cases and 20 million skeletal fluorosis cases in 17 countries [12]. A recent meta-analysis of published studies suggests that more work needs to be done on investigating the possibility of an adverse effect of high fluoride exposure on children's neurodevelopment [13]. The toxic effects of fluoride are a continuous concern.

Drinking water is usually the main source of fluoride. Depending on age the relative source contribution varies between 41% and 71% [14]. A study in Iran showed that the contribution of drinking water to total fluoride exposure can range from 70%–90%, depending on the level of fluoride in the drinking water [15]. Concern about elevated fluoride levels in drinking water is not based so much on acute toxicity effects, but rather on long-term consumption of high-fluoride drinking water. Many epidemiological studies have shown the positive relationship between the fluoride concentration of drinking water and the prevalence and severity of dental fluorosis [16]. In Estonia this relationship was found to be very strong (*r* = 0.93) and that allowed calculation of the risk of dental fluorosis for the different levels of fluoride in drinking water [17].

Information about fluoride content in drinking water makes it is possible to estimate the population exposed to elevated levels of fluoride. Previous research conducted in Estonia determined that 4.1% of the population was at risk of exposure to elevated fluoride in drinking water [18]. This risk can be prevented or minimized in one or more of several ways. 

Fluoride is a chemical compound very difficult to remove from water. A number of investigations have been made on a variety of treatment methods for the removal of fluoride. These methods can be broadly divided into two categories: membrane techniques, including reverse osmosis, nanofiltration, dialysis and electro-dialysis; and the adsorption technique, which is a conventional technique applying adsorbents such as alumina/aluminum based materials, clays and soils, calcium based minerals, synthetic compounds and carbon-based materials [19].

There are mainly two recognized methods to remove fluoride from water in public water systems: reverse osmosis filtration and activated alumina defluoridation. The selection of treatment process should be site specific as each technology has own advantages and limitations [20].

The other ways to reduce the fluoride level in public water supply are using alternative water sources with a suitable fluoride level, mixing water from different sources (dilution with low-fluoride sources) *etc*. The best solution depends on local (hydrogeological) circumstances. There is no unique method that is suitable for every situation.

The purpose of current study was to follow the changes in excessive fluoride exposure of population on country level and to analyze the methods used by public water supplies (PWS) to reduce the fluoride levels in water under local circumstances.

## 2. Materials and Methods

### 2.1. Study Area

Estonia, with an area of 45,227 km^2^ and population of 1.294 million people (1 January 2012), is the smallest Baltic country. Hydrogeologically Estonian sedimentary rocks form a typical artesian basin, where five aquifer systems are used for drinking water purposes. The drinking water supply is based mainly on groundwater. In two towns, capital Tallinn and Narva, the water supply is based mainly on surface water. Estonia is characterized by a large number of water supplies due to quite low population density (29.8 inhabitants/km^2^). Only about 5% of PWS serve more than 2,000 inhabitants. The main toxic chemical of health concern is fluoride which is a naturally occurring chemical element in some groundwater layers.

### 2.2. Data and Methods

This study is a longitudinal follow-up study where the changes in fluoride content in drinking water were measured every four years during the period of 2004–2012. The study base was a country-wide study of all drinking water supplies serving at least 100 inhabitants and covering 82.9% of Estonian population. Water samples were taken from 47 towns and 471 rural settlements. The detailed description of the study and methods used are published elsewhere [21,22]. For current study only public water supplies providing high-fluoride (>1.5 mg/L) drinking water were selected for the follow-up (104 out of 518). These PWS were informed about the undesirable results in 2004 and recommended to implement measures to reduce the excessive content of fluoride in water. Two follow-ups were performed. In 2008, all PWS registered with high fluoride content in a 2004 study were re-visited and water samples taken according to the same methodology used in 2004. Also, information was collected from the PWS which methods have been implemented to reduce the fluoride content. In the last follow-up in 2012, the fluoride concentration in PWS was obtained from Health Board of Estonia who is responsible for the surveillance of drinking water quality of all PWS. Also, the public water suppliers were contacted to obtain data about applied fluoride reduction measures during 2008–2012.

The PWS sizes were categorized as follows: (1) up to 200; (2) 201–500; (3) 501–1,000; (4) 1,001–2,000; (5) over 2,000 consumers. The fluoride remediation measures that were implemented in PWS to reduce the fluoride content in the system were grouped into four categories:

(1) Water treatment (reverse osmosis)
(2) New water source (construction of a new well)
(3) Joining another (larger) water system in other town or region
(4) Other PWS reconfiguration measures (disconnecting non-compliant wells from the supply system, water mixing with another wells with lower fluoride content, *etc.*)

The population exposure was measured by linking the data of fluoride concentration in each PWS and their corresponding served population. In case of uncertainties, the PWS and local municipalities were consulted with. Population exposure to excessive fluoride drinking water (over 1.5 mg/L) was divided into four exposure intensity categories:

(1) 1.51–2.0 mg/L – low intensity
(2) 2.1–3.0 mg/L   – moderate intensity
(3) 3.1–4.0 mg/L   – medium intensity
(4) > 4.0 mg/L    – high intensity

## 3. Results

The results of this study give an overview of reduction of excessive exposure to drinking water fluoride among Estonian population on a country level and implemented measures during 2004–2012.

### 3.1. Population Exposure to Excessive Drinking Water Fluoride

**Figure 1:**
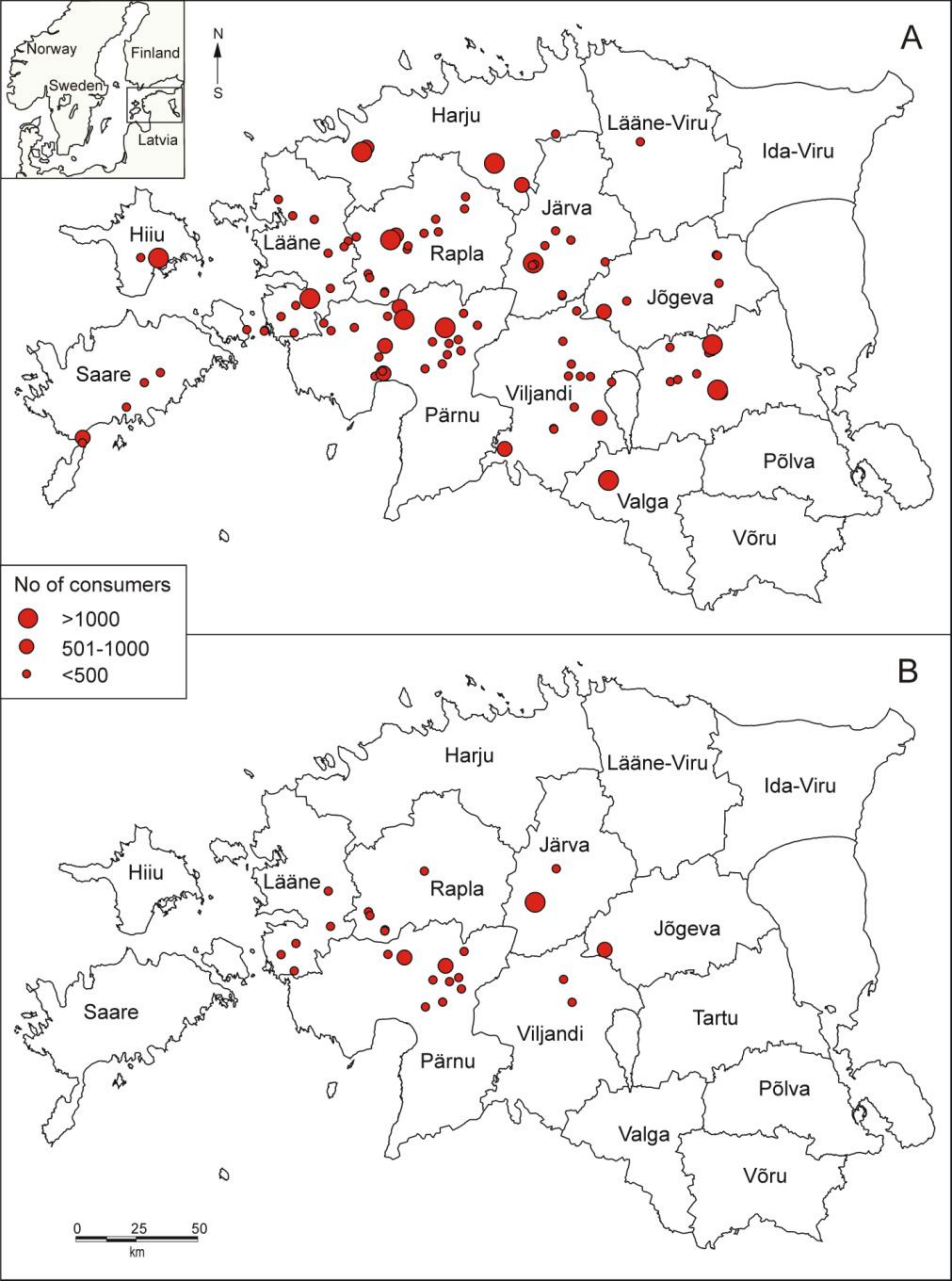
Map of the study area and location of public water supplies with high (>1.5 mg/L) fluoride content by the size of PWS in 2004 (**A**) and 2012 (**B**).

In 2004 high-fluoride drinking water was provided by 104 public water supply systems. In total 42,571 inhabitants consumed water with high fluoride content (Table 1). This amounted to 4.1% of the Estonian population. High-fluoride PWS were located mostly located in the central and western parts of Estonia, in southern and northern parts only few PWS extracted high fluoride water (Figure 1A). The majority (77.9%) of these PWS were small (up to 500 consumers), and this counts less than half of the excessive exposure (41.8%). There were only two large PWS (over 2,000 consumers) (1.9%). These PWS made up to 10.7% of excessive exposure. The exposure intensity in large PWS was mainly low (up to 2.0 mg/L). Consumers in high intensity exposure category formed only 9.3% of exposed population. They were mainly consumers of small PWS (83.3%).

By 2008 the fluoride content was reduced to acceptable (up to 1.5 mg/L) levels in 38 (36.5%) water supplies, including two large water supplies (over 2,000 consumers). As the result, the overall exposure to excessive fluoride was decreased by 19,834 consumers (46.6%). According to the analysis of exposure reduction by intensity categories the highest decrease (58.9%) was found among high exposure intensity group (F > 4.0 mg/L). Also, in low intensity group, the exposure was decreased by half (50.0%). Altogether, there were still 22,737 inhabitants in 2008 consuming high fluoride drinking water from 66 PWS, most of them (72.7%) were very small water supplies.

In 2012 there were 25 PWS, which were non-compliant with the fluoride regulations. These PWS are located mostly in western Estonia in rural areas (Figure 1B). 60% of these PWS are small-sized (up to 200 consumers) and 24% small-sized (201–500 consumers) PWS. These two categories make up to 48.2% of exposure. Only one PWS is quite large (1,600 consumers), and accounts for 1/5 of the exposure (20.9%). Population exposed to high fluoride drinking water was 7,673 inhabitants and the reduction in exposure was 82.0% as compared to year 2004. High intensity exposure was still experienced by 380 inhabitants (5.0%).

**Table 1:** Exposure to high fluoride drinking water by intensity category and size of public water supply (PWS) in 2004, 2008 and 2012.

	2004		2008		2012	
	No of PWS	No of consumers	No of PWS	No of consumers	No of PWS	No of consumers
**Intensity category** (concentration of fluoride in drinking water, mg/L)						
1.51–2.0	37	22,392	26	11,184	12	4,856
2.1–3.0	41	10,626	22	6,024	8	1,089
3.1–4.0	14	5,613	10	3,909	3	1,348
>4.0	12	3,940	8	1,620	2	380
Total	104	42,571	66	22,737	25	7,673
**PWS size by no of consumers**						
up to 200	46	6,135	35	4,832	15	1,946
201–500	35	11,650	20	6,614	6	1,749
501–1,000	11	7,091	6	4,365	3	2,378
1,001–2,000	10	13,125	5	6,926	1	1,600
>2,000	2	4,570	0	0	0	0
Total	104	42,571	66	22,737	25	7,673

### 3.2. Fluoride Reduction Measures Implemented by PWS

The reduction of fluoride in tap water was achieved due to efforts of public water suppliers. Several mitigation measures were used by PWS to reduce the excessive level of fluorides in tap water. The selection and implementation of methods depended on local geographical, hydrogeological factors and financial resources. Four type of measures were used: water treatment by reverse osmosis, finding alternative water source (new well), joining PWS to other one with better water quality; and reconfiguration of PWS (water mixing *etc*.). Measures were undertaken in 85 PWS, the remaining 19 had not made any changes. In case of six PWS the implemented method and positive results were not continuous, *i.e.*, the fluoride concentration was not reduced to the desirable level or had increased again after some time period. That was confirmed in three PWS using reverse osmosis treatment and in three PWS using use new water sources. 

As a result of remediation measures, the overall exposure to excessive fluorides was reduced from 42,571 to 35,210 (82.7%) inhabitants by 2012 (Table 2). One third of exposure reduction (33.8%) was achieved by using alternative water sources (25 PWS). The reverse osmosis technique was implemented at 26 PWS (exposure reduction 25.6%). Twenty PWS had joined another PWS with lower fluoride content and this reduced the exposure among 21.7% of exposed inhabitants. 

**Table 2:** Measures implemented to reduce fluoride content in drinking water during 2004–2012 in Estonia.

Measure	PWS		Consumers	
	No	%	No	%
Reverse osmosis	26	30.6	8,998	25.6
New water source (well)	25	29.4	11,913	33.8
Connection to another PWS	20	23.5	7,634	21.7
Other reconfiguration measures	14	16.5	6,665	18.9
Total	85	100	35,210	100

**Figure 2:**
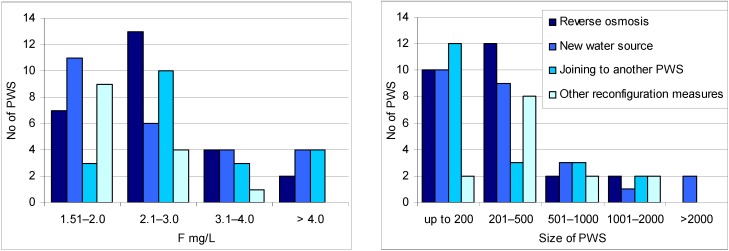
Implemented measures by the size of PWS and concentration of fluoride (exposure intensity) in drinking water.

The distribution of implemented measures by the size of PWS and the content of fluoride in water (exposure intensity) are presented in Figure 2.

## 4. Discussion

This study produced an estimate of reduction in population exposure to the high-fluoride drinking water and fluoride reduction measures implemented in 2004–2012 on the basis of country-wide data. A three-step study was carried out. The first step was undertaken in 2004 and comprised an intensive tap water sampling program for fluoride in all public water supplies (PWS) serving 100 or more consumers to obtain the data for the exposure of population to different levels of fluoride from drinking water [21,22]. The water sampling covered 93.7% of the population having access to public water supplies. In this paper we included data only on PWS having high content of fluoride (over 1.5 mg/L) in water. Two follow-up studies were carried out in a four year interval (in 2008 and 2012) to monitor the PWS with high fluoride drinking water about the water quality and measures taken to reduce undesirable exposure. Fluoride content in drinking water is only one element in assessing population exposure. A second element is the number of people exposed to elevated fluoride concentrations. In addition, exposure also depends on the volume of fluoride-rich water consumed and also the amount of fluoride obtained from elsewhere in the diet. In our study we only analyzed the fluoride exposure through drinking water as the main source. Data on water consumers of PWS was recalculated for every study period to take into account population mobility.

The provision of a safe supply of drinking water is the most important prerequisite for a healthy life. In Estonia the responsibility for water supply lies on local administrations. In 2004 the overall access to PWS was 82.9%. This is substantially higher than the global average (56%), but similar to other Baltic States [23]. The variations in access between urban and rural population as well as geographical regions are significant. Due to dispersed allocation of population (29.8 inhabitants/km^2^) small PWS are dominant in Estonia. Up to 86% of PWS serve less than 500 inhabitants [18]. That complicates the improvement and inspection of water quality and makes it expensive.

In Estonia, naturally-occurring fluoride is the main chemical of health concern in drinking water. There is no industry or human activity that can cause anthropogenic pollution of water with fluoride. The high levels of fluoride in groundwater originate from geogenic sources [4,5,7]. The hydro-geochemical studies of groundwater make it possible to delimit the fluoride anomaly (up to 7.2 mg/L) in western Estonia. The source of fluoride-rich groundwater is the dissolution of fluorides from carbonate rocks and clayey K-bentonite beds, mainly in the Silurian-Ordovician aquifer system [6]. 

In 2004 high-fluoride water was discovered in 104 groundwater-based PWS and 42,571 inhabitants were estimated to experience excessive fluoride exposure through drinking water. Majority of these PWS (78%) were small (up to 500 consumers) and located mainly in western and central Estonia, which coincides with the distribution area of the Silurian-Ordovician aquifer system [3].

Exceeding the limit value of 1.5 mg/L was a breach of national standard, and PWS had to undertake measures to fulfill the requirements. A transition period was given to PWS to achieve the standard until 2007. Reducing the naturally high levels of fluoride in water is difficult and expensive. The best solution depends on the local circumstances, both technical and economical.

The first option is to find an alternative source of water with a suitable fluoride level. These sources include surface water and low-fluoride groundwater. Surface water is usually prone to contamination with biological and chemical pollutants and cannot be used for drinking purposes without treatment and disinfection. It makes this source too expensive and complex for application in small and poor municipalities. For small PWS the low-fluoride groundwater would be preferable. The hydro-geological conditions determine the resources, availability and protection against pollution of the source. Also, mixing water from different sources can lower the fluoride level in drinking water. De-fluoridation of drinking water is the only practicable option to overcome the problem of excessive fluoride in drinking water in regions, where alternate source is not available. The techniques available for de-fluoridation include membrane process, ion exchange, coagulation-precipitation and adsorption processes. The membrane process, mainly the reverse osmosis technique, is used, but it requires high maintenance cost due to fouling, scaling, and degradation of membrane. Similarly, the ion exchange process is very costly. Although coagulation-precipitation is an effective and cheap method, its main disadvantage is the generation of harmful waste products [19].

In our study, where high-fluoride drinking water was the problem in small rural communities, the reverse osmosis or replacement of water sources with new ones (mainly new drilled wells) was the first option. Reverse osmosis was more often applied in western Estonia, where the Silurian-Ordovician high-fluoride aquifer system is the only source of freshwater. It should be mentioned, that the method is costly and requires careful maintenance of the supply system. In many cases the desired effect was achieved as a result of the PWS joining a bigger PWS from a neighboring town or settlement. In this case the quality and control of water is guaranteed. In the PWS using several sources (drilled wells) that differ in terms of fluoride content in water, it was possible to mix water from wells to obtain better quality.

As the result of implemented measures the exposure to high-fluoride drinking water was reduced substantially (82%). How rapidly and to what extent measures are implemented in remaining high-fluoride PWS depends on local conditions and financial resources.

It is very important to provide information on the levels of fluoride in drinking water to the public health professional, water companies and also to the general population. The results of current study are continuously disseminated through Health Board of Estonia. Information about fluoride content in drinking water is made available to public health professionals (incl stomatologists) in order to plan oral health strategies, as well as to the local water supply companies.

## 5. Conclusions

The population exposure to high-fluoride drinking water during 2004–2012 has decreased considerably. The reduction of fluoride in tap water is due to efforts of public water suppliers. The renovation of water system has been a priority in Estonia and most of the actions have been done with the financial support of EU mechanisms.

The optimization of fluoride levels was achieved by different methods. Water treatment by reverse osmosis or replacement of water sources with new ones (mainly new drilled wells) were the most frequently used measures. Reverse osmosis is the only possibility in some rural regions where there is only one water source with high natural fluoride content.

Regular monitoring of tap water quality is still needed to guarantee the proper operation of the water treatment. Joining small PWS to larger ones with good water quality as well as reconfiguration of PWS has provided desirable effect.
